# Assessment of activation delay in the right ventricular outflow tract as a potential complementary diagnostic tool for Brugada Syndrome

**DOI:** 10.1093/europace/euaf093

**Published:** 2025-06-13

**Authors:** Cinzia Monaco, Ivan Eltsov, Alvise Del Monte, Filippo Aglietti, Luigi Pannone, Domenico Della Rocca, Anaïs Gauthey, Antonio Bisignani, Sahar Mouram, Paul-Adrian Calburean, Gudrun Pappaert, Gezim Bala, Antonio Sorgente, Alexandre Almorad, Erwin Stroker, Juan Sieira, Andrea Sarkozy, Gian Battista Chierchia, Mark La Meir, Pedro Brugada, Carlo de Asmundis

**Affiliations:** Heart Rhythm Management Centre, Postgraduate Program in Cardiac Electrophysiology and Pacing, European Reference Networks Guard-Heart, Vrije Universiteit Brussel, Universitair Ziekenhuis, Laarbeeklaan 101, Brussel 1090, Belgium; Cardiac Surgery Department, Vrije Universiteit Brussel, Universitair Ziekenhuis Brussel, Brussel, Belgium; Heart Rhythm Management Centre, Postgraduate Program in Cardiac Electrophysiology and Pacing, European Reference Networks Guard-Heart, Vrije Universiteit Brussel, Universitair Ziekenhuis, Laarbeeklaan 101, Brussel 1090, Belgium; Heart Rhythm Management Centre, Postgraduate Program in Cardiac Electrophysiology and Pacing, European Reference Networks Guard-Heart, Vrije Universiteit Brussel, Universitair Ziekenhuis, Laarbeeklaan 101, Brussel 1090, Belgium; Heart Rhythm Management Centre, Postgraduate Program in Cardiac Electrophysiology and Pacing, European Reference Networks Guard-Heart, Vrije Universiteit Brussel, Universitair Ziekenhuis, Laarbeeklaan 101, Brussel 1090, Belgium; Heart Rhythm Management Centre, Postgraduate Program in Cardiac Electrophysiology and Pacing, European Reference Networks Guard-Heart, Vrije Universiteit Brussel, Universitair Ziekenhuis, Laarbeeklaan 101, Brussel 1090, Belgium; Heart Rhythm Management Centre, Postgraduate Program in Cardiac Electrophysiology and Pacing, European Reference Networks Guard-Heart, Vrije Universiteit Brussel, Universitair Ziekenhuis, Laarbeeklaan 101, Brussel 1090, Belgium; Heart Rhythm Management Centre, Postgraduate Program in Cardiac Electrophysiology and Pacing, European Reference Networks Guard-Heart, Vrije Universiteit Brussel, Universitair Ziekenhuis, Laarbeeklaan 101, Brussel 1090, Belgium; Heart Rhythm Management Centre, Postgraduate Program in Cardiac Electrophysiology and Pacing, European Reference Networks Guard-Heart, Vrije Universiteit Brussel, Universitair Ziekenhuis, Laarbeeklaan 101, Brussel 1090, Belgium; Heart Rhythm Management Centre, Postgraduate Program in Cardiac Electrophysiology and Pacing, European Reference Networks Guard-Heart, Vrije Universiteit Brussel, Universitair Ziekenhuis, Laarbeeklaan 101, Brussel 1090, Belgium; Heart Rhythm Management Centre, Postgraduate Program in Cardiac Electrophysiology and Pacing, European Reference Networks Guard-Heart, Vrije Universiteit Brussel, Universitair Ziekenhuis, Laarbeeklaan 101, Brussel 1090, Belgium; Heart Rhythm Management Centre, Postgraduate Program in Cardiac Electrophysiology and Pacing, European Reference Networks Guard-Heart, Vrije Universiteit Brussel, Universitair Ziekenhuis, Laarbeeklaan 101, Brussel 1090, Belgium; Heart Rhythm Management Centre, Postgraduate Program in Cardiac Electrophysiology and Pacing, European Reference Networks Guard-Heart, Vrije Universiteit Brussel, Universitair Ziekenhuis, Laarbeeklaan 101, Brussel 1090, Belgium; Heart Rhythm Management Centre, Postgraduate Program in Cardiac Electrophysiology and Pacing, European Reference Networks Guard-Heart, Vrije Universiteit Brussel, Universitair Ziekenhuis, Laarbeeklaan 101, Brussel 1090, Belgium; Heart Rhythm Management Centre, Postgraduate Program in Cardiac Electrophysiology and Pacing, European Reference Networks Guard-Heart, Vrije Universiteit Brussel, Universitair Ziekenhuis, Laarbeeklaan 101, Brussel 1090, Belgium; Heart Rhythm Management Centre, Postgraduate Program in Cardiac Electrophysiology and Pacing, European Reference Networks Guard-Heart, Vrije Universiteit Brussel, Universitair Ziekenhuis, Laarbeeklaan 101, Brussel 1090, Belgium; Heart Rhythm Management Centre, Postgraduate Program in Cardiac Electrophysiology and Pacing, European Reference Networks Guard-Heart, Vrije Universiteit Brussel, Universitair Ziekenhuis, Laarbeeklaan 101, Brussel 1090, Belgium; Heart Rhythm Management Centre, Postgraduate Program in Cardiac Electrophysiology and Pacing, European Reference Networks Guard-Heart, Vrije Universiteit Brussel, Universitair Ziekenhuis, Laarbeeklaan 101, Brussel 1090, Belgium; Cardiac Surgery Department, Vrije Universiteit Brussel, Universitair Ziekenhuis Brussel, Brussel, Belgium; Heart Rhythm Management Centre, Postgraduate Program in Cardiac Electrophysiology and Pacing, European Reference Networks Guard-Heart, Vrije Universiteit Brussel, Universitair Ziekenhuis, Laarbeeklaan 101, Brussel 1090, Belgium; Heart Rhythm Management Centre, Postgraduate Program in Cardiac Electrophysiology and Pacing, European Reference Networks Guard-Heart, Vrije Universiteit Brussel, Universitair Ziekenhuis, Laarbeeklaan 101, Brussel 1090, Belgium

**Keywords:** Brugada syndrome, ECGi, Activation time, Diagnostic tool

## Abstract

**Aims:**

In patients with Brugada syndrome (BrS), diagnosis relies primarily on the presence of the characteristic type 1 electrocardiographic (ECG) pattern. The aim of this study was to propose an alternative diagnostic method in situations where ECG alone is uncertain.

**Methods and results:**

This study was conducted in two phases: (i) Phase 1: cut-off determination. Controls and BrS patients were analysed to develop a predictive model based on electrocardiographic imaging (ECGi) parameters for the diagnosis of BrS. Patients with right bundle branch block (RBBB) were analysed separately. All patients underwent ajmaline infusion. Concealed BrS patients were evaluated in both the absence and presence of a type 1 ECG pattern. The right and left ventricular ‘epicardium’ maps obtained with ECGi were divided into eight regions, and the mean activation time (ATm) was calculated for each region. The ATm for each area was normalized to QRS length (ATm%); ATm and ATm% were compared across populations. (ii) Phase 2: cut-off validations. A new cohort of control and BrS patients was used to perform a blinded validation of the proposed method. In Phase 1 (cut-off determination), 57 patients affected by BrS, and 10 controls were included. Analysis of ATm and ATm% in right ventricular outflow tract (RVOT) showed significant differences between controls and BrS patients both with either concealed or manifested Pattern 1 ECG (3 721 ± 6.23 vs. 68.33 ± 14.73 ms, *P* < 0.001; 37.21 ± 6.23 vs. 107.57 ± 21.16 ms, *P* < 0.001). The relationship between the anterior-RV and the RVOT ATm was used to develop a predictive model to identify a diagnostic threshold for BrS diagnosis. An increase of 45% in anterior-RV ATm was determined to be the optimal predictor of delayed RVOT activation in BrS patients (area under the receiver operating characteristic curve = 0.97, accuracy = 0.92, *F*-score = 0.95). In RBBB patients, the ATm delay cut-off was reached exclusively in cases with concomitant BrS. In Phase 2, 7 out of 7 control patients exhibited a percentage increase between the anterior-RV and RVOT of <45%. Among BrS patients with concealed pattern (pattern-concealed), 11 out of 20 showed a percentage increase >45% (accuracy 67%). In BrS patients with manifested Pattern 1 (pattern-positive), 19 out of 20 showed a percentage increase of >45% (accuracy 96%).

**Conclusion:**

In BrS, the delay in RVOT activation can be identified using a threshold value of 45% above the mean activation time in the anterior-RV for each patient, offering a reliable diagnostic tool when standard ECG method alone falls short.

## Graphical Abstract

**Graphical Abstract:**
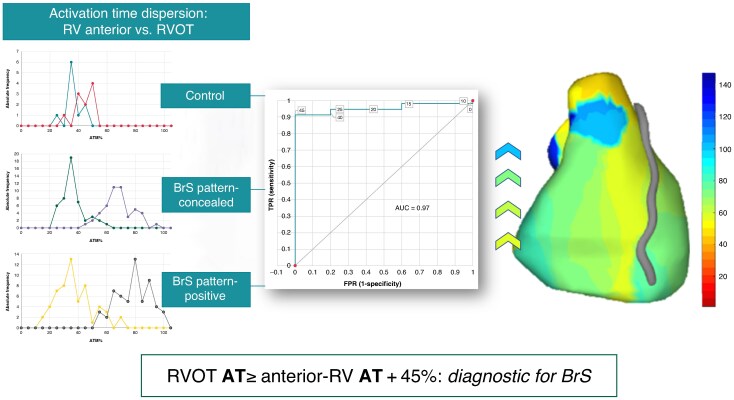

**What Is New?**

- In a single patient, an epicardial ECGi activation delay in the right ventricular outflow tract (RVOT) exceeding 45% compared to his own anterior right ventricular (anterior-RV) activation may indicate the presence of Brugada syndrome (BrS).
- This method has the potential to improve BrS diagnosis in situations where an ECG alone is uncertain or not feasible, such as in cases of bundle branch block, allergies, or hypersensitivity to specific medications, or in the presence of overlapping syndromes that may worsen with pharmacological induction.

## Introduction

Even 30 years after its discovery, the debate surrounding Brugada syndrome (BrS) remains open, with ongoing discussions on its aetiopathogenesis, risk stratification, and treatment strategies.^1,2^ Over the past decade, histopathological studies have raised new questions regarding the nature of the disease.^3,4^ Initially considered a condition occurring in completely healthy hearts, it is now recognized as a disease affecting macrostructurally healthy heart,^5^ prompting some authors to differentiate between healthy individuals with a Brugada pattern and patients suffering from Brugada syndrome.^6,7^ While many aspects of the syndrome have evolved, one aspect that has remained unchanged over the past 30 years is the diagnosis, which relies on detecting the type 1 electrocardiographic (ECG) pattern, either spontaneously or drug-induced,^8–10^ as a sufficient criterion^8^ or in combination with other clinical features.^11^ Therefore, while the presence of Pattern 1 is not necessarily indicative of the disease and does not necessarily constitute the presence of an arrhythmogenic substrate (indeed, in the presence of the pattern, malignant ventricular arrhythmias do not always develop^12^). Pattern 1 is reflective of unique electrophysiological (EP) characteristics in BrS, especially (but not exclusively) at the level of the anterior aspect of the right ventricular outflow tract (RVOT).^13–17^ Despite its critical role in diagnosis, relying solely on ECG can sometimes lead to uncertainties, as distinguishing the BrS pattern from other ECG morphologies can be challenging. Additionally, the use of pharmacological agents to unmask the pattern in patients with concealed ECG may be limited by concomitant diseases, unavailability of the drugs, or hypersensitivity to these agents.^18–22^

The purpose of this study was to investigate the relationship between the presence of BrS ECG Pattern 1 and the BrS EP characteristics using electrocardiographic imaging (ECGi), to provide additional diagnostic support for BrS in cases where standard ECG methods alone are uncertain.

## Methods

This study was conducted in two phases, integrating patient cohorts from the Universitair Ziekenhuis of Brussel (UZB). Phase 1 (Cut-off determination): this involved retrospective analysis of ECGi data from a BrS patient cohort, in addition to a group of control subjects and a group of patients having right bundle branch block (RBBB). Phase 2 (Validation): In this phase, the proposed cut-off was blindly validated using prospectively enrolled and independent cohorts from UZB, divided similarly to Phase 1. The aim of Phase 2 was then to assess the cut-off's diagnostic performance.

The exclusion of macrostructural heart diseases was performed in both populations by transthoracic echocardiogram, computed tomography (CT), and/or magnetic resonance imaging (MRI). All patients signed informed consent that was approved by our institutional review board. All data regarding BrS patients were collected and updated in the monocentric registry of the UZB (NCT05283759). The study complied with the Declaration of Helsinki as revised in 2013; the ethics committee approved the study.

### BrS diagnosis

The BrS was diagnosed according to current recommendations.^23^ Ajmaline (1 mg/kg) was administered intravenously over a 5-min period to unmask the diagnostic pattern in cases of concealed baseline ECG.^24^ The drug infusion was discontinued if QRS prolongation exceeded 30%, frequent premature ventricular beats or type I Brugada ECG occurred, or due to the development of high degree atrio-ventricular block.^10^

**Figure 1:**
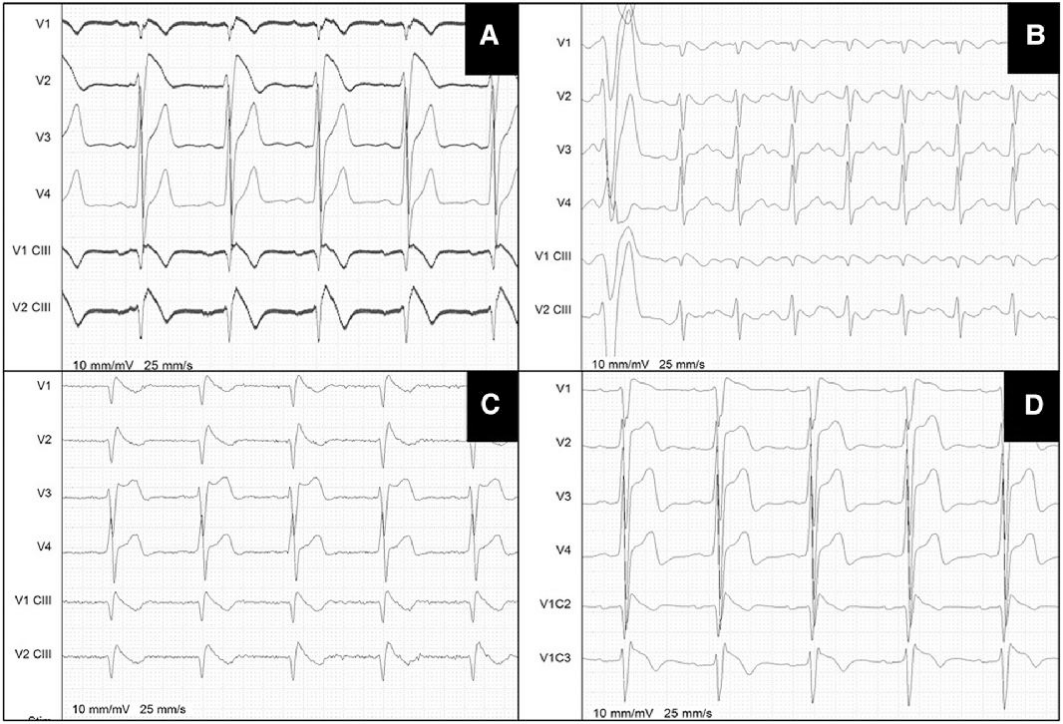
Four patients diagnosed with BrS in Phase 1 of the study, after pharmacological induction. All ECGs were evaluated by an expert operator and all patients achieved a Shanghai score ≥ 3.5 points. The baseline ECGs are shown in Supplementary material online, *Figure S1* Supplementary Materials. *(A)* Classic ECG presentation of BrS pattern in leads V2 and V2-third intercostal space. *(B)* ECG with initially uncertain interpretation. A BrS pattern is present in leads V1 and V1-third intercostal space (coved ST segment, T-wave inversion), but the leads exhibit microvoltage, and the J-point elevation does not reach 2 mm above the isoelectric line. The PVC at the start of the ECG trace (also recorded with standard lead positions) originates from the anterior RVOT. *(C)* ECG with initially uncertain interpretation. The ST segment in the right precordial leads appears steep and lacks the classic convexity associated with the BrS pattern. *(D)* ECG with initially uncertain interpretation. The patient exhibited a baseline incomplete right bundle branch block (RBBB). Only during the washout phase of the pharmacological provocation test did the ST segment in lead V2-second intercostal space exhibit a coved morphology (as shown), whereas it remained concave during the peak of drug induction. BrS, Brugada syndrome; RVOT, right ventricular outflow tract.

**Figure 2:**
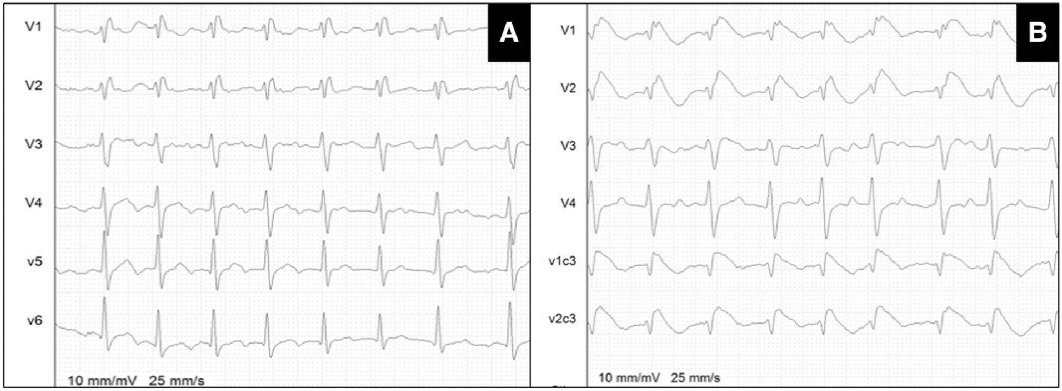
Example of a patient with baseline RBBB *(A)* and BrS pattern at the peak of the pharmacological provocation test *(B)*.

In cases of borderline ECG pattern morphology (*Figure 1* and Supplementary material online, *Figure S1*), RBBB (*Figure 2*), or in instances of disagreement between two operators, the diagnosis was determined based on the opinion of an expert operator (C.d.A. or P.B.) and/or with a Shanghai score ≥ 3.5.^25^

### Phase 1: patient characteristics

As mentioned, three groups of patients were included in Phase 1: (i) patients diagnosed with BrS and (ii) control patients were used to build the diagnostic tool. In control patients, BrS was investigated due to familiar screening, compatible symptoms, BrS patterns 2 or 3 on the ECG, and excluded with drug provocation test. (iii) Patients with baseline RBBB were analysed separately, categorized into patients having RBBB only (RBBB-controls) and patients with concomitant RBBB and BrS (RBBB-BrS). All patients with RBBB also underwent pharmacological provocation testing with ajmaline.

All patients underwent ECGi and ECG as part of the study protocol. Clinical risk stratification was calculated using the score system developed at UZB by Sieira *et al*.^26^ Genetic analysis for BrS was performed with Roche SeqCap® EZ Human Exome Probes v3.0.^27^

### Phase 2: patient characteristic

This validation cohort consisted of patients diagnosed with BrS and control patients. All patients underwent ECGi and ECG as part of the study protocol. For this phase of the study, all data were anonymized and independently collected by two operators (I.E. and L.P.), with discrepancies reconciled as needed. A third physician (C.M.) performed the analysis, diagnosing BrS patients in a blinded manner if their score exceeded the cut-off identified in the first phase of the study, while classifying all other patients as negative. The diagnoses of BrS or non-BrS were subsequently compared with the gold standard, which was based on the diagnoses provided by the center’s physicians and validated by an expert operator (C.d.A or P.B.), applying the same criteria used in Phase 1 to distinguish BrS and control patients. Accuracy metrics were then calculated for the validation population.

### Electrocardiographic imaging

The ECGi methodology has been described previously^28–30^ (Supplementary material online, *Figure S2*). Briefly, all patients had the 252 electrodes of the CardioInsight Noninvasive 3D Mapping System technology (Medtronic Inc, Minneapolis, MN, USA) positioned on their chest. A computed tomography 120 kV scan protocol with high-resolution 64 slices CT Revolution scan system (GE Healthcare, IL, USA) was used to acquire the images. Segmentation of the CT scan was performed, creating a detailed three-dimensional (3D) shell of the heart—biventricular for our purpose. The CT scan was then merged with ECGi electrodes using automatic software on the CardioInsight workstation, and 3D geometry was subsequently modelled to add the atrioventricular valves and left anterior descending coronary artery. Unipolar electrograms derived from the 252 electrodes placed on the chest were projected onto the 3D geometry, and maps of activation time (AT), repolarization time (RT), activation-repolarization interval (ARI) were automatically obtained. Local AT (referenced to the beginning of QRS in ECG lead II) was determined by the maximal negative slope of the electrograms (EGMs) during QRS inscription, as previously described.^31^ Local RT was determined from the maximum derivative of the EGM T-wave.^32^ Local ARI was defined as the difference between RT and AT.

### Post-processing analysis tool

**Figure 3:**
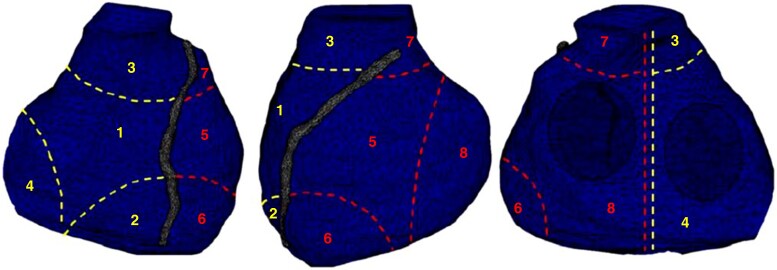
The CT scan was used to capture images of the heart, and the epicardial surface was divided into 4 regions for each ventricle. The left anterior descendent (LAD) coronary artery served as a reference to distinguish the right ventricle (RV) and the left ventricle (LV). The regions were numbered 1–8, with regions 1–4 corresponding to the RV and regions 5–8 to the LV: (1) anterior RV, (2) RV apex, (3) RV outflow tract (RVOT), (4) posterior-lateral RV, (5) anterior LV, (6) LV apex, (7) LV outflow tract (LVOT), and (8) posterior-lateral LV.

A novel tool was designed at UZB for the study purposes with MatLab (version 7.10.0 Natick, MA, USA: The MathWorks Inc.; 2010), enabling the post-processing analysis of ECGi maps in the Phase 1. The tool had the advantage of sampling an entire area of the ventricular epicardial surface to allow a macroscopic assessment of the EP parameters, which are not susceptible to changes in local action potential. Regional EP parameter, including mean AT (ATm), mean RT (RTm), and mean activation-repolarization interval (ARIm), were automatically calculated, eliminating outliers from each measurement, to avoid over-distortion caused by epicardial gradients between adjacent electrodes. The regions were created by sampling the area of the biventricular epicardium (in square centimetres), with the anatomical boundaries identified as previously described^29,31^ (*Figure 3* and Supplementary material online, *Figure S3*). ATm, RTm, and ARIm were analysed as raw values and were then normalized for QRS length (ATm%, RTm%, ARIm%). Every measure was repeated for 3 consecutive beats. On average, approximately 12 000 EGMs per patient were obtained for analysis.

On the other hand, measurements in the Phase 2 groups were taken by averaging 3 points in the targeted cardiac zones, for 3 consecutive beats, without using the post-processing tool, to simulate the real conditions of application.

### Statistical analysis

All variables were tested for normality with the Shapiro–Wilk test. Normally distributed variables were described as mean ± standard deviation, and the groups were compared through paired or unpaired *t*-tests as appropriate, while non-normally distributed variables were described as median (interquartile range) and compared by the Mann–Whitney test or the Wilcoxon signed-rank test as appropriate. Categorical variables were described as frequencies (percentages) and compared by the χ2 test, Fisher exact test, or ANOVA test as appropriate. All statistical tests were performed at the level of ‘epicardial’ segments. Differences in variables among ‘epicardial’ segments were compared by 1-way repeated-measures analysis of variance. Using the Bonferroni method, pairwise comparisons between RVOT and anterior right ventricle (anterior-RV) were conducted. For the ATm analysis, the values were derived from the mean AT for each area over three consecutive beats and were normalized for the QRS length of each patient (ATm%). Increases in ATm and ATm% were calculated between anterior-RV and RVOT for each population, and the data were distributed on a relative and absolute frequency distribution, considering progressive steps of 5%. In the validation study, the cut-off was applied blindly to the population and validation was done with an accuracy study. Sensitivity and specificity were calculated at the best cut-off derived by Youden's method. Discrimination was measured by the area under the receiver operating characteristic curve measure (AUC). A *P* value <0.05 was considered statistically significant. The analysis was performed using R software version 3.6.2 (R Foundation for Statistical Computing, Vienna, Austria).

## Results

### Phase 1: cut-off determination

A total of 77 participants (mean age, 46.38 ± 24.54 years; 45 men), including 64 diagnosed with BrS and 13 patients from the control group, were enrolled in the study. Seven patients from the BrS group and 3 from the control group presented with a RBBB and were therefore analysed separately.

Among BrS population, 48 out of 57 patients (84.21%) had a concealed ECG at baseline (BrS pattern-concealed), while the remaining 9 patients (15.78%) exhibited a spontaneous Pattern 1 ECG (BrS pattern-spontaneous). Patients with concealed BrS were evaluated both under baseline conditions and also during drug-induced Pattern 1 (BrS pattern-induced). Patients with a spontaneous or drug-induced type I ECG did not show significant difference regarding ECG features and EP parameters (see Supplementary material online, *Table S1*) and were then grouped together (BrS pattern-positive). Patients’ characteristics are included in *Table 1*.

**Table 1:** Patient characteristics in Phase 1 of the study. The BrS pattern-positive group includes patients who exhibited pattern 1 ECG after the ajmaline test (BrS pattern-induced) as well as 9 patients who presented with a spontaneous pattern 1 at baseline (BrS pattern-spontaneous)

	BrS (57)		Controls (10)
	BrS pattern- concealed (48)	BrS pattern-positive (pattern-induced and pattern-spontaneous) (57)	
Age, y	38 ± 14	39 ± 14	55 ± 18
Male sex, (*n*, %)	24 (50%)	31 (54%)	8 (80%)
Syncope (*n*, %)	19 (40%)	24 (42%)	1 (10%)
Aborted SCD (*n*, %)	2 (4%)	4 (7%)	0
SCD family history (*n*, %)	8 (17%)	10 (17%)	2 (20%)
VA inducibility at EPS (*n*, %)	4 (8%)	6 (10%)	N.A.
Shanghai score (points)^25^	4.3 ± 1.0	4.3 ± 1.0	1.7 ± 0.8
Sieira score (points)^26^	3.1 ± 2.2	3.4 ± 2.3	0.6 ± 1.1
ICD (*n*, %)	21 (44%)	27 (47%)	0
SND (*n*, %)	8 (17%)	10 (17%)	1 (10%)
Positive genetic test, *n* pt (%)	16 (33%)	23 (40%)	N.A.
Ajmaline administered (mg)	67 ± 14	N.A.	73 ± 8
Ajmaline percentage of target dose (%)	91 ± 14	N.A.	100
ECG QRS, mean (ms)	101 ± 11	143 ± 19	88 ± 8
ECG QRS right precordial leads, mean (ms)	103 ± 13	153 ± 20	88 ± 7
ECG PQ, mean (ms)	149 ± 13	171 ± 30	146 ± 13
Beta-angle right precordial leads, median [IQR]^33^	24° [18°−32°]	N.A.	16° [11°−19°]

BrS, Brugada syndrome; EPS, electrophysiological study; ICD, implantable cardioverter defibrillator; SCD, sudden cardiac death; SND, sinus node disfunction; VA, ventricular arrhythmia.

**Figure 4:**
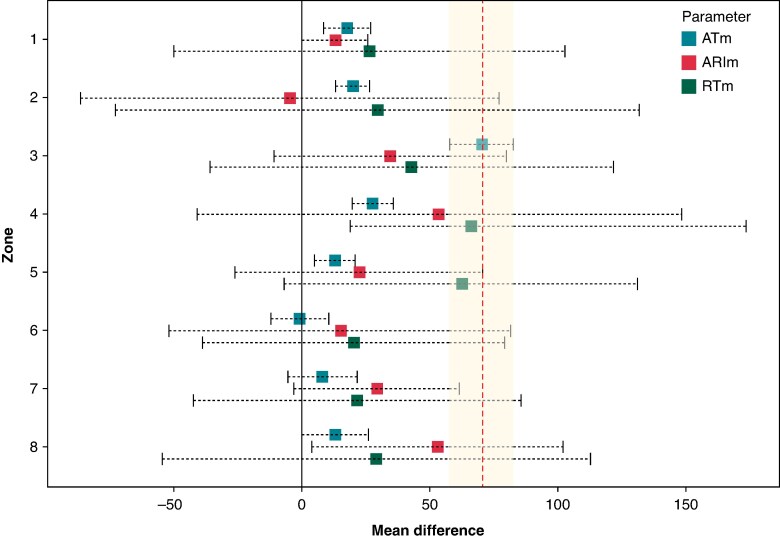
Mean differences and 95% confidence interval (CI) of mean activation times (ATm), mean activation-repolarization intervals (ARIm), and mean repolarization times (RTm) between BrS pattern-positive patients and the control group, recorded via ECGi and divided by cardiac zones. The greatest difference with the narrowest dispersion (95% CI range) corresponds to ATm in zone 3 (RVOT). The 95% CI of ATm does not include zero in 6 out of 8 zones and are in general the narrowest, confirming AT as the parameter with the highest statistical precision among those available. Zone 1 (anterior-RV) was chosen as a reference, as it shows the lowest variability in ATm, ARIm, and RTm compared with all other zones and exhibits the earliest ATm both in BrS and controls, as shown in Supplementary material online, *Table S2a* of the supplementary materials. Zone 1: anterior-RV; 2: RV apex; 3: RVOT; 4: posterior-lateral RV; 5: anterior LV; 6: LV apex; 7: LVOT; 8: posterior-lateral LV. ARIm, activation repolarization time; ATm, activation time, mean; BrS pattern-positive, BrS patients during manifested ECG pattern 1; RTm, repolarization time, mean.

The ATm, RTm, and ARIm were measured in both BrS and control groups (see Supplementary material online, *Table S2A*). These EP parameters were systematically compared across cardiac zones to identify the one that showed the greatest power to differentiate BrS patients from controls (see Supplementary material online, *Table S2B*). Among them, ATm in the RVOT demonstrated the largest mean difference between the two groups (ATm_BrS_RVOT vs. ATm_control_RVOT: 70.3 ms; 95% CI: 57.9–82.8; *P* < 0001; *Figure 4*) and was therefore selected as the most discriminative parameter between controls and BrS patients.

**Figure 5:**
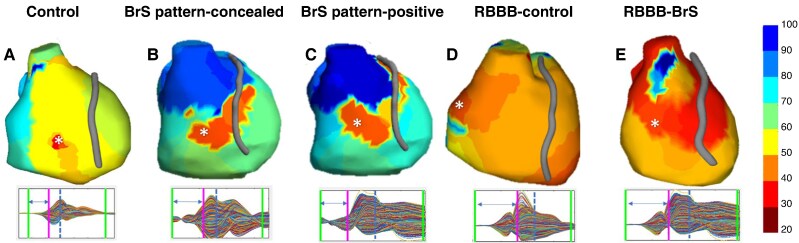
Post-processed AT maps generated using the MatLab tool for the Phase 1 study populations. The first epicardial breakthrough is indicated with asterisks. The bottom panels display the window of interest for the electrogram recordings. The blue line represents the -dV/dT, and the purple line indicates the annotation of the first breakthrough. BrS, Brugada syndrome; BrS pattern-concealed, BrS patients with concealed ECG; BrS pattern-positive, BrS patients during manifested ECG pattern 1; RBBB, right bundle branch block.

To correctly assess this RVOT activation delay, we measured its prolongation from the anterior-RV, which showed the earliest ATm in both BrS pattern-positive patients and controls, and was the least sensitive to variation in the RV between these two populations (Supplementary material online, *Figure* S*4*). The anterior-RV area was identified by the post-processing tool within the designated anatomical region (Zone 1), which included the first breakthrough area (indicated by an asterisk in *Figure 5*). Further details on the first breakthrough are provided in the Supplementary Materials. The activation gradient between anterior-RV and RVOT was calculated as both an absolute and percentage increase and used to establish a delayed activation cut-off that correlated with the presence of BrS.

The values of ATm and ATm% for Zone 1 (anterior-RV) and Zone 3 (RVOT) are listed in *Table 2*.

**Table 2:** Phase 1: values of ATm and ATm% in control group and BrS, and their increase between anterior-RV and RVOT (absolute and percentage). Only patients with BrS (BrS pattern-concealed and BrS pattern-positive) have an increase greater than the study-determined pathological cut-off of 45% and are shown in bold

**Controls**	**Controls (10)**			
	**anterior-RV, ATm (ms)**	**RVOT, ATm (ms)**	**Increase (ms)**	**Increase%**
	30.72 ± 4.01	37.21 ± 6.23	6.49 ± 7.23	21.13%±13.66
	**anterior-RV, ATm%**	**RVOT, ATm%**	**Increase (ms)**	**Increase%**
	34.22 ± 5.14	41.25 ± 6.29	7.03 ± 8.13	20.54%±11.71
**BrS**	**BrS pattern-concealed (48)**			
	**anterior-RV, ATm (ms)**	**RVOT, ATm (ms)**	**Increase (ms)**	**Increase%**
	34.89 ± 11.23	68.33 ± 14.73	33.44 ± 18.64	**95.84%±66.91**
	**anterior-RV, ATm%**	**RVOT, ATm%**	**Increase (ms)**	**Increase%**
	34.11 ± 8.31	65.79 ± 10.39	31.68 ± 11.28	**92.88%±40.73**
	**BrS pattern-positive (57)**			
	**anterior-RV, ATm (ms)**	**RVOT, ATm (ms)**	**Increase (ms)**	**Increase%**
	53.30 ± 19.41	107.57 ± 21.16	54.27 ± 25.13	**101.82%±39.62**
	**anterior-RV, ATm%**	**RVOT, ATm%**	**Increase (ms)**	**Increase%**
	36.10 ± 14.82	70.09 ± 11.77	33.99 ± 15.55	**94.16%±44.17**

anterior-RV, anterior right ventricle; ATm, activation time, mean; BrS, Brugada Syndrome; BrS pattern-concealed, BrS patients with concealed ECG; BrS pattern-positive, BrS patients during manifested ECG pattern 1; RBBB, right bundle branch block; RVOT, right ventricle outflow tract.

### Activation times in anterior RV

Analysis of anterior-RV ATm did not show differences between controls and BrS pattern-concealed patients (30.72 ± 4.01 vs. 34.98 ± 11.22 ms, *P* = 0.24), while there was a difference between controls and BrS pattern-positive patients (30.72 ± 4.01 vs. 55.28 ± 19.42 ms, *P* < 0001). Analysis of ATm% did not show differences between the control group and BrS patients, either in the absence or presence of Pattern 1 ECG (controls vs. BrS pattern-concealed: 34.18 ± 5.13 vs. 34.08 ± 8.27 ms, *P* = 0.97; controls vs. BrS pattern-positive: 34.18 ± 5.13 vs. 35.07 ± 12.82 ms, *P* = 0.83).

### Activation times in RVOT

Analysis of RVOT ATm revealed significant differences between controls and BrS patients, both in the absence and presence of pattern 1 ECG (controls vs. BrS pattern-concealed: 37.21 ± 6.23 vs. 68.33 ± 14.73 ms, *P* < 0001; controls vs. BrS pattern-positve: 37.21 ± 6.23 vs. 107.57 ± 27.16 ms, *P* < 0001). Similarly, ATm% showed significant differences (controls vs. BrS pattern-concealed: 41.25 ± 6.29 vs. 65.79 ± 10.39 ms, *P* < 0001; controls vs. BrS pattern-positive: 41.25 ± 6.29 vs. 70.09 ± 11.77 ms, *P* < 0001).

**Figure 6:**
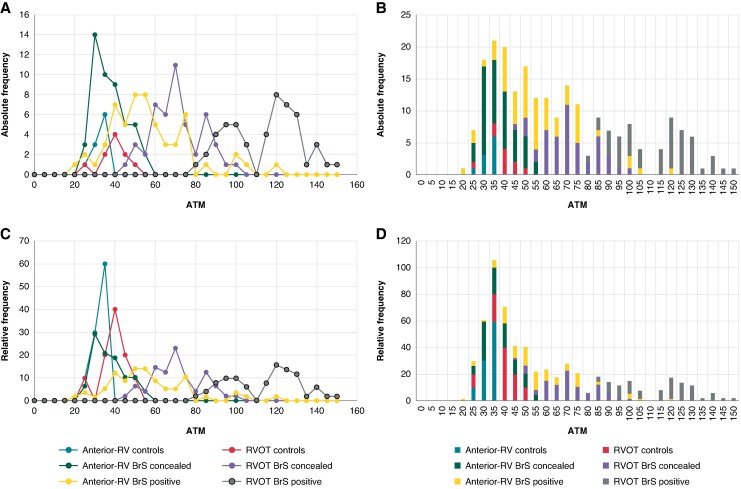
Distribution of ATm by absolute (*A* and *B*) and relative (*B* and *C*) frequency. The scatter plots (*A* and *C*) show that the anterior RV and RVOT ATm values for the control group are concentrated in the first half of the graph, while in BrS patients, values are dispersed in the right part of the graph, particularly for RVOT values in BrS pattern-positive patients. The stacked bar charts (*B* and *D*) indicate that the median ATm values are around 40 ms, with values exceeding 100 ms observed exclusively in the BrS + population. anterior-RV, right ventricle anterior; ATm, activation time mean; BrS, Brugada syndrome; BrS pattern-concealed, BrS patients with concealed ECG; BrS pattern-positive, BrS patients during manifested ECG pattern 1; RVOT, right ventricle outflow tract.

**Figure 7:**
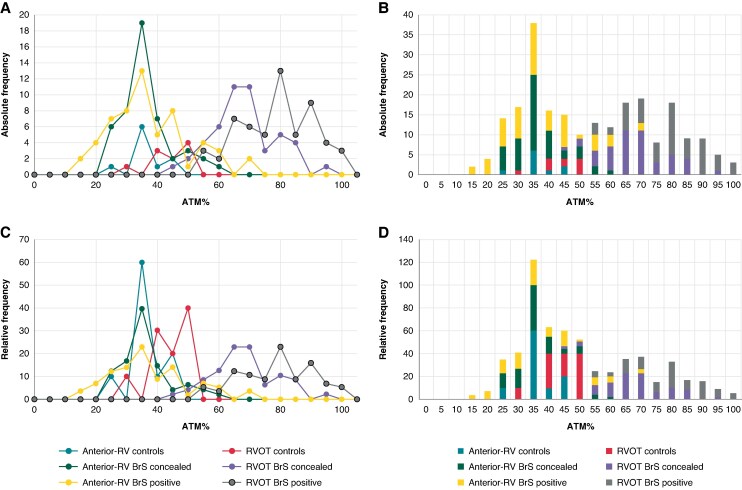
Distribution of ATm% by absolute (*A* and *B*) and relative (*C* and *D*) frequency. The scatter plots (*A* and *C*) demonstrate that the ATm values normalized to QRS duration are less dispersed compared with the previous figure, showing two areas of concentration: in the first part of the graph (including all anterior-RV ATm% and RVOT ATm% values from the control group) and in the second part (where only RVOT ATm% values from the BrS population are found). The stacked column graphs (*B* and *D*) illustrate that all ATm% values fall within 60% of QRS duration, except for RVOT values in BrS patients and a small percentage of RV anterior values in the BrS pattern-positive population, where values are mostly above 60% of QRS length. anterior-RV, right ventricle anterior; ATm%, activation time mean%; BrS, Brugada syndrome; BrS pattern-concealed, BrS patients with concealed ECG; BrS pattern-positive, BrS patients during manifested ECG pattern 1; RVOT, right ventricle outflow tract.

The distributions of ATm and ATm% values are shown in *Figures 6* and *7*.

### RVOT activation delay prediction model

**Figure 8:**
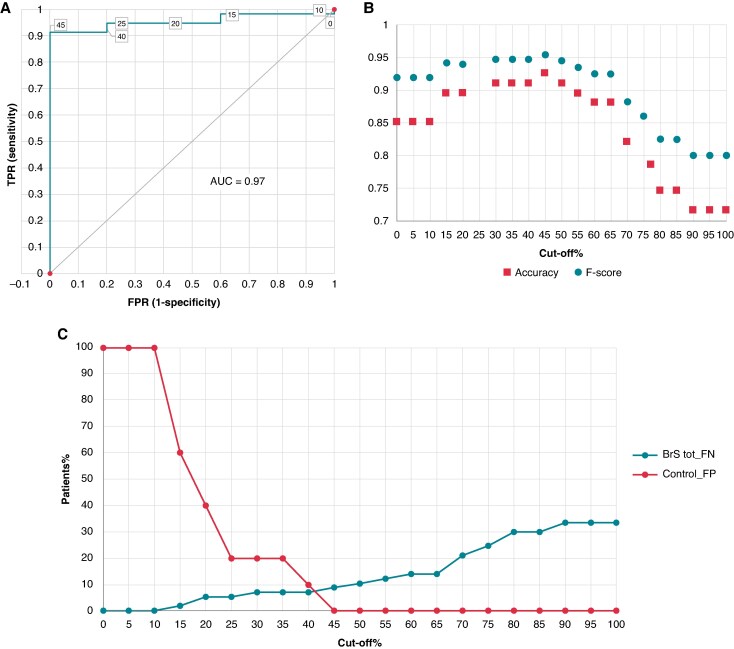
Diagnostic performance tests. *(A)* The ROC curve shows the trade-off between the true positive rate and the false positive rate at different classification thresholds, represented by the percentage increase in ATm from the anterior RV to the RVOT. A 45% cut-off corresponds to 100% specificity and >90% sensitivity (AUC = 0.97). *(B)* A 45% increase in ATm from the anterior RV to the RVOT yields the highest accuracy and F-score. *(C)* The x-axis represents the cut-off values as the percentage increase in ATm from the RV anterior to the RVOT. The two curves refer to false positives in the total BrS population and false negatives in the control population. As can be seen, the two curves intersect above 40%, and at 45%, the false positive population is equal to zero. BrS, Brugada syndrome; FN, false negative; FP, false positive; FPR, false positive rate; TPR, true positive rate.

*Table 2* shows the relative increase of ATm and ATm% between anterior-RV and RVOT within control and BrS populations. Setting the activation delay between anterior-RV and RVOT as a cut-off, and considering the controls as the true negative population and the BrS pattern-positive patients as the true positive population, diagnostic performance tests were done to identify the best value that could discriminate in the total population the BrS, as shown in *Figure 8*. By dividing the percentage delay increment into steps of 5%, the two curves intersect above 40%, and at 45% the false positives are zero. The diagnostic performance test attested that a 45% increase in anterior-RV ATm is the best cut-off of delayed RVOT activation in BrS patients (AUC = 0.97; accuracy = 0.92; *F*-score = 0.95), and this is not influenced by the QRS duration (ATm%).

In a single patient, an activation delay in the RVOT >45% compared with his own anterior-RV activation could indicate the presence of Brugada syndrome.

### Activation delay in RBBB patients

Three patients from the control group and seven from the BrS population presented with RBBB. For all the RBBB patients at baseline, the ATm in the anterior-RV was delayed compared with controls (RBBB vs. controls: 67.61 ± 10.31 ms vs. 30.72 ± 4.01 ms, *P* < 0.001) and was instead similar to BrS pattern-positive patients (RBBB vs. BrS pattern-positive: 67.61 ± 10.31 ms vs. 55.28 ± 19.42 ms, *P* = 0.078). Analysis of ATm% showed a significant delay in the anterior-RV of RBBB patients compared with both controls and BrS patients (RBBB vs. controls: 45.16 ± 7.05% vs. 34.18 ± 5.13%, *P* < 0.001; RBBB vs. BrS pattern-positive: 45.16 ± 7.05% vs. 35.07 ± 12.82%, *P* = 0.01).

Regarding the RVOT, both ATm and ATm% differed significantly from controls (ATm RBBB vs. controls: 119.83 ± 22.03 ms vs. 37.21 ± 6.21 ms, *P* < 0.001; ATm%: 80.29 ± 16.10% vs. 41.25 ± 6.29%, *P* < 0.001) but were similar to BrS patients (ATm RBBB vs. BrS pattern-positive: 119.83 ± 22.03 ms vs. 130.36 ± 21.69 ms, *P* = 0.16; ATm%: 80.29 ± 16.10% vs. 86.96 ± 15.93%, *P* = 0.22).

Splitting the RBBB population into RBBB-controls and RBBB-BrS, the ATm and ATm% were similar in the anterior-RV (ATm RBBB-controls vs. RBBB-BrS: 66.54 ± 8.55 ms vs. 68.07 ± 13.08 ms, *P* = 0.86; ATm%: 45.07 ± 7.15% vs. 45.66 ± 8.82%, *P* = 0.92) but the ATm differed significantly in the RVOT (ATm RBBB-controls vs. RBBB-BrS: 95.29 ± 14.48 ms vs. 130.36 ± 21.69 ms, *P* = 0.03; ATm%: 64.74 ± 12.57% vs. 87.96 ± 15.93%, *P* = 0.056).

The increase between anterior-RV and RVOT in RBBB-controls did not reach the cut-off of 45%, whereas it did for RBBB-BrS, as reported in Supplementary material online, *Table S3*.

### Phase 2

#### Cut-off validation

A total of 27 patients (mean age, 44 ± 17 years; 17 men), including 20 diagnosed with BrS and 7 control group patients, were enrolled in the validation cohort. Among the BrS population, all patients had a concealed ECG at baseline and were studied under baseline conditions (BrS pattern- concealed) and during drug-induced type 1 ECG (BrS pattern-positive).

In the control group, all patients (7 out of 7, 100%) exhibited a percentage ATm increase between the anterior-RV and RVOT (both at baseline and after ajmaline testing) of <45%. In BrS pattern- concealed patients, 11 out of 20 (55%) showed a percentage increase >45% [sensitivity 55%, specificity 100%, positive predictive value (PPV) 100%, negative predictive value (NPV) 44%, accuracy 67%]. In BrS pattern-positive, 19 out of 20 (95%) showed a percentage increase >45% (sensitivity 95%, specificity 100%, PPV 100%, NPV 87.5%, accuracy 96%). An overview of the results for the Phase 2 populations is shown in *Table 3*.

**Table 3:** Phase 2: values of ATm and ATm% in control group and BrS, and their increase between anterior-RV and RVOT (absolute and percentage). Only patients with BrS have an increase greater than the study-determined pathological cut-off of 45%, that is shown in bold

**Controls**	**Controls (7)**			
	**Anterior-RV, ATm (ms)**	**Anterior-RV, ATm (ms)**	**Increase (ms)**	**Increase%**
	31.04 ± 5.65	37.82 ± 5.76	6.78 ± 7.79	21.84 ± 15.78
	**Anterior-RV, ATm%**	**Anterior-RV, ATm%**	**Increase (ms)**	**Increase%**
	36.66 ± 6.47	42.01 ± 7.25	5.35 ± 9.65	14.59 ± 11.16
**BrS**	**BrS pattern-concealed (20)**			
	**Anterior-RV, ATm (ms)**	**Anterior-RV, ATm (ms)**	**Increase (ms)**	**Increase%**
	32.25 ± 7.25	53.37 ± 20.68	21.12 ± 19.72	**65.49** **±** **37.73**
	**Anterior-RV, ATm%**	**Anterior-RV, ATm%**	**Increase (ms)**	**Increase%**
	35.22 ± 7.62	59.24 ± 22.96	24.02 ± 24.17	**68.20** **±** **32.85**
	**BrS pattern-positive (20)**			
	**Anterior-RV, ATm (ms)**	**Anterior-RV, ATm (ms)**	**Increase (ms)**	**Increase%**
	48.45 ± 18.12	118.15 ± 33.86	69.70 ± 38.33	**143.86** **±** **79.17**
	**Anterior-RV, ATm%**	**Anterior-RV, ATm%**	**Increase (ms)**	**Increase%**
	40.43 ± 15.06	98.45 ± 28.29	58.02 ± 31.94	**143.51** **±** **76.63**

Anterior-RV, anterior right ventricle; ATm, activation time, mean; BrS, Brugada Syndrome; BrS pattern-concealed, BrS patients with concealed ECG; BrS pattern-positive, BrS patients during manifested ECG pattern 1; RBBB, right bundle branch block; RVOT, right ventricle outflow tract.

## Discussion

This study introduces a potential alternative diagnostic method for Brugada syndrome in cases where the ECG alone is uncertain or borderline. By employing a cut-off based on a 45% delay in RVOT activation relative to the anterior-RV, diagnostic performance tests demonstrated high accuracy and reliability, highlighting the potential for enhancing BrS diagnosis. In cases where ECG alone is insufficient (e.g. due to RBBB) or cannot be performed (e.g. due to allergies or hypersensitivity to class IA/IC antiarrhythmics, unavailability of these drugs, coexistence of overlapping syndrome that may worsen with pharmacological induction^18^) identifying a complementary diagnostic method based on patient-specific electrophysiological parameters, rather than a fixed cut-off, may open new avenues for diagnosis. Furthermore, to the best of our knowledge, this is the largest ECGi study of the BrS population to date. The aim of this study, therefore, was not to demonstrate the presence of a pathological entity at the level of the RVOT, but rather to leverage the potential mechanism underlying the development of the type 1 pattern—specifically, the activation delay occurring in the RVOT—as an integrative diagnostic method. Building on the complexities outlined, we included an illustration of a ‘textbook’ BrS Pattern 1 ECG in *Figure 1A*, contrasted with ECGs in *Figure 1B, C, and D* that feature less distinct characteristics. These cases exemplify the diagnostic ambiguities often encountered in clinical practice. Despite the subtler presentations, these patients were validated as having BrS, displaying typical characteristics of a pattern 1 (delayed activation of the right sections, negative T wave, ST segment elevation) with a Shanghai score over 3.5. This score reflects clinical backgrounds such as SCN5A mutations, affected first-degree relatives, syncope, and/or documented ventricular arrhythmias, after ruling out all other possible causes.

The other key findings of this research are as follows: (i) Although BrS-related abnormalities can extend beyond the RVOT, RVOT activation time was the parameter with the most variability (higher mean difference) between BrS patients and controls (*Figure 3*); (ii) The RV showed greater differences in EP parameters between BrS patients and controls compared with the LV, particularly in activation times. The anterior-RV showed the lower difference compared with healthy controls in the whole RV. Notably, significant differences in the anterior-RV wall were only observed in BrS patients during Pattern 1; on the contrary, RVOT variations were significant both with and without Pattern 1. (iii) In patients with RBBB, the ATm in the anterior-RV is similar to BrS pattern-positive, but the delay in RVOT ATm exceeds the 45% cut-off only in BrS patients.

### BrS activation Time

The macroregional analysis of ATm aligns with previous studies on BrS.^29^ Compared with control group, there is a significant difference in terms of activation in the anterior-RV of BrS patients with Pattern 1 ECG. However, this finding does not hold when ATm is normalized for QRS duration (ATm%), where no difference is observed between controls and BrS patients, regardless of the presence of Pattern 1. The difference in ATm, but not in the ATm%, may indicate a global conduction delay associated with BrS pattern, rather than a delay specific to the anterior-RV.

Another notable observation is that anterior-RV activation is delayed in patients with RBBB compared with BrS pattern-concealed patients, but similar between RBBB patients and BrS pattern-positive patients, having patients with RBBB and BrS Pattern 1 a similar QRS duration (QRS BrS pattern-positive vs. QRS RBBB: 152.99 ± 20.09 vs. 150 ± 12.76, *P* = 0.6). However, when normalized for QRS duration, ATm in RBBB patients was significantly greater than that in BrS pattern-positive patients, indicating that in BrS, the anterior-RV activation delay is part of a global conduction slowing. Conversely, in RBBB patients, even after normalization, the delay remains significantly longer, due to the intrinsic conduction defect.

Unlike the anterior-RV, ATm in the RVOT consistently showed a delay in BrS patients, independent of QRS duration and the presence of Pattern 1 (ATm and ATm% in controls vs. BrS pattern- concealed and controls vs. BrS pattern-positive: *P* < 0.001). Prior studies have demonstrated substantial changes in RVOT electrophysiology, regardless of the presence or absence of Pattern 1.^15^ This allows for the establishment of a cut-off for pathological RVOT activation delay, further discussed in the next section.

### RVOT activation delay prediction model

Diagnostic performance tests identified that an increase in anterior-RV ATm of >45% detects pathological RVOT activation delay in BrS patients. These findings suggest that RVOT activation time delay is effective for identifying BrS in 95% of patients with Pattern 1 and in over half of BrS patients even in the absence of the pattern (Phase 2). Furthermore, this delay is not associated with the drug used to induce the BrS ECG pattern or with an intrinsic conduction defect, as demonstrated by controls who underwent the ajmaline test and RBBB patients, where the anterior-RV to RVOT delay cut-off was not reached.

Previous studies have already demonstrated the role of conduction disturbance in BrS. For example, late potentials (LP) have been identified as a noninvasive marker strongly correlated with life-threatening events in BrS patients.^34^ Electroanatomical mapping studies have further confirmed that BrS patients exhibit prolonged RV activation, increased electrogram fractionation, and delayed transmural conduction, providing evidence for a conduction substrate in BrS independent of repolarization abnormalities.^35^

The use of ECGi to detect delays in RVOT activation complements earlier studies by providing a noninvasive and macroregional assessment of the conduction delays that characterize BrS. Our findings reinforce the role of ‘epicardial’ non-contact mapping in detecting alterations in the RVOT, as previously described.^15,16,36^ This methodology has predominantly characterized the BrS pattern through its ability to capture conduction delays specific to the RVOT region.^37^

Letsas *et al*.^38^ have similarly demonstrated prolonged RVOT activation time in BrS patients through bipolar and unipolar electroanatomic contact mapping (BrS vs. controls: 86.4 ± 16.5 ms vs. 63.4 ± 9.7 ms, *P* < 0.001), aligning with our results and further validating the use of RVOT activation delay as a diagnostic parameter.

#### BrS and RBBB

Distinguishing between BrS ECG pattern and RBBB can sometimes be challenging.^19^ Moreover, where the two conditions coexist, unmasking the BrS pattern may not always be feasible using standard ECG techniques.^39^ In this study, three patients from the control group and seven from the BrS cohort presented with RBBB. Based on the identified cut-off, the increases in ATm and ATm% between the anterior-RV and RVOT in RBBB patients were analysed. The increase in the anterior-RV to RVOT delay for RBBB-controls remained below the 45% cut-off, while it exceeded 45% in the RBBB-BrS population, allowing for differential diagnosis between these two populations.

#### Limitations

This study includes BrS patients both with and without spontaneous or drug-induced type 1 patterns. Most BrS patients presented a high-risk profile (Phase 1: Sieira score 3.37 ± 2.33, ICD 47.36%; Phase 2: Sieira score 2.56 ± 1.97, ICD 35%) because, for ethical reasons, CT scans—required for ECGi—are primarily performed on patients with a higher risk of arrhythmic events. However, in the Phase 2 cohort, some low-risk patients were also included, with promising results. Future studies should differentiate between low-risk and high-risk BrS populations, as the pathological substrate may be less pronounced or even absent in the low-risk group and, therefore, may not display the same electrophysiological characteristics.

The sample sizes of RBBB patients and controls are smaller compared with the BrS group. However, we compared our findings with previous studies that used the same populations and methodology.^29,40^ Despite the limited data, our results for these populations align with the findings reported in the literature.

A key limitation of this study is the absence of testing for the functional nature of the conduction block, which may lead to false positives, as activation delay is not specific to BrS and can be attributed to various pathologies. While this limitation was not directly addressed in the main analysis, it is important to acknowledge it as a potential constraint of the proposed diagnostic approach.

## Conclusions

Patients with Brugada syndrome exhibit a significantly delayed activation time in the right ventricular outflow tract, measurable relative to the activation time of the anterior right ventricle —the region of the right ventricle less affected by AT variability. The threshold distinguishing normal conduction from BrS-associated delay can be identified by adding 45% to the mean activation time in the anterior RV for each patient. This method can serve as a complementary diagnostic tool and may provide a potential support in cases where conventional ECG-based diagnosis is challenging or uncertain.

## Supplementary Material

euaf093_Supplementary_Data

## Data Availability

The data underlying this article will be shared on reasonable request to the corresponding author.
