# Supplementary material for: Assessment of activation delay in the right ventricular outflow tract as a potential complementary diagnostic tool for Brugada Syndrome
Source: Europace. 2025 Jun 13;27(6):euaf093. doi: 10.1093/europace/euaf093 (PMC12164293; doi:10.1093/europace/euaf093)
Supplement: euaf093_Supplementary_Data [file euaf093_supplementary_data.docx]

### Supplementary Materials

**Table 1 Supplementary Materials.** The table includes the entire population of patients with Brugada Syndrome in the Phase 1 of the study, with both ECG pattern induced pharmacologically (BrS pattern-induced, 48 patients) or spontaneously (BrS pattern-spontaneous, 9 patients). The two populations did not show differences, with the exception of the values reported in bold; for the purposes of the study, the populations have therefore been treated as a single population (BrS pattern-positive: 57 patients).

|  | **BrS pattern-induced (48)** | **BrS pattern-spontaneous (9)** | **Total BrS pattern-positive (57)** | **p value** |
| --- | --- | --- | --- | --- |
| **Age, y** | 38±14 | 44±10 | 39±14 | 0,24 |
| **Male sex, (n, %)** | 24 (50%) | 7 (78%) | 31 (54%) | 0,21 |
| **History of syncope (n, %)** | 19 (39%) | 5 (55%) | 24 (42%) | 0,6 |
| **Aborted SCD (n, %)** | 2 (4%) | 2 (22%) | 4 (7%) | 0,21 |
| **SCD family history (n, %)** | 8 (17%) | 2 (22%) | 10 (17%) | 0,93 |
| **SND (n, %)** | 8 (17%) | 2 (22%) | 10 (17%) | 0,93 |
| **VA inducibility at EPS (n, %)** | 4 (8,33%) | 2 (22,22%) | 6 (10%) | 0,51 |
| **Sieira score (points)** | 3,07±2,25 | 4,33±2,44 | 3,37±2,33 | 0,13 |
| **ICD (n, %)** | 21 (44%) | 6 (67%) | 27 (47%) | 0,36 |
| **Positive genetic test, n pt (%)** | 16 (33%) | 7 (78%) | 23 (40%) | **0,03** |
| **ECG QRS right precordial leads, mean (ms)** | 153±20 | 149±10 | 153±20 | 0,57 |
| **ECG PQ, mean (ms)** | 167±25 | 192±34 | 171±30 | **0,01** |
| **ATm anterior-RV (ms)** | 52,60±20,03 | 63,45±16,37 | 55,28±19,42 | 0,13 |
| **ATm% anterior-RV (ms)** | 34,06±13,03 | 42,23±10,2 | 35,07±12,82 | 0,12 |
| **ATm RVOT (ms)** | 107,28±29,09 | 107,6±21,16 | 107,57±27,16 | 0,97 |
| **ATm% RVOT (ms)** | 70,90±12,61 | 72,27±12,95 | 70,09±11,77 | 0,56 |

ATM: activation time, mean; BrS: Brugada Syndrome; ICD: implantable cardioverter defibrillator; SCD: sudden cardiac death; SND: sinus node disfunction; RV: right ventricle; RVOT: right ventricle outflow tract.

**Table 2a Supplementary Materials.** Mean activation times (Ats), activation-repolarization intervals (ARIs) and repolarization times (RTs) for study populations (Phase 1).

1: anterior RV; 2: RV apex; 3: RVOT; 4: posterior-lateral RV; 5: anterior LV; 6: LV apex; 7: LVOT; 8: posterior-lateral LV.

| **Parameters** | | **Zones** | | | | | | | |
| --- | --- | --- | --- | --- | --- | --- | --- | --- | --- |
|  |  | **1** | **2** | **3** | **4** | **5** | **6** | **7** | **8** |
| **Controls (10)** | **Mean AT, ms** | 31±4 | 34±3 | 37±6 | 35±5 | 46±8 | 52±7 | 55±6 | 62±11 |
| **BrS+ (57)** | **Mean AT, ms** | 53±19 | 58±11 | 107±22 | 60±16 | 57±8 | 56±13 | 70±23 | 77±13 |
| **Controls (10)** | **Mean ARI, ms** | 232±45 | 245±65 | 239±34 | 234±39 | 233±43 | 234±31 | 244±61 | 246±49 |
| **BrS+ (57)** | **Mean ARI, ms** | 234±91 | 249±78 | 294±91 | 245±55 | 243±65 | 267±46 | 286±59 | 279±78 |
| **Controls (10)** | **Mean RT, ms** | 242±56 | 257±74 | 290±66 | 241±59 | 278±66 | 266±68 | 269±77 | 281±73 |
| **BrS+ (57)** | **Mean RT, ms** | 267±93 | 261±81 | 333±99 | 275±78 | 286±55 | 273±32 | 292±54 | 302±45 |

ARI: activation-repolarization interval; AT: activation time; BrS: Brugada Syndrome; LV: left ventricle; LVOT: left ventricular outflow tract; RT: repolarization time; RV: right ventricle; RVOT: right ventricular outflow tract.

| **Table 2b Supplementary Materials.** Mean ATs, ARIs and RTs for study populations (Phase 1), paired for each zone.  1: anterior RV; 2: RV apex; 3: RVOT; 4: posterior-lateral RV; 5: anterior LV; 6: LV apex; 7: LVOT; 8: posterior-lateral LV.  Paired Samples T-Test | | | | | | | | | | | | | | | | | | | | | | | |
| --- | --- | --- | --- | --- | --- | --- | --- | --- | --- | --- | --- | --- | --- | --- | --- | --- | --- | --- | --- | --- | --- | --- | --- |
|  | | | | | | | | | | | | | | | | **95% Confidence Interval** | | | |  | | | |
|  | |  | |  | | **Statistic** | | **df** | | **p** | | **Mean difference (ms)** | | **SE difference** | | **Lower** | | **Upper** | |  | | **Effect Size** | |
| AT_BrS_1 |  | AT_control_1 |  | Student's t |  | 4.402 |  | 9.00 |  | 0.002 |  | 17.809 |  | 4.05 |  | 8.657 |  | 27.0 |  | Cohen's d |  | 1.3920 |  |
| AT_BrS_2 |  | AT_control_2 |  | Student's t |  | 6.788 |  | 9.00 |  | < .001 |  | 19.865 |  | 2.93 |  | 13.245 |  | 26.5 |  | Cohen's d |  | 2.1467 |  |
| AT_BrS_3 |  | AT_control_3 |  | Student's t |  | 12.831 |  | 9.00 |  | < .001 |  | 70.349 |  | 5.48 |  | 57.947 |  | 82.8 |  | Cohen's d |  | 4.0576 |  |
| AT_BrS_4 |  | AT_control_4 |  | Student's t |  | 7.873 |  | 9.00 |  | < .001 |  | 27.812 |  | 3.53 |  | 19.820 |  | 35.8 |  | Cohen's d |  | 2.4895 |  |
| AT_BrS_5 |  | AT_control_5 |  | Student's t |  | 3.695 |  | 9.00 |  | 0.005 |  | 12.891 |  | 3.49 |  | 4.998 |  | 20.8 |  | Cohen's d |  | 1.1684 |  |
| AT_BrS_6 |  | AT_control_6 |  | Student's t |  | -0.141 |  | 9.00 |  | 0.891 |  | -0.703 |  | 4.98 |  | -11.958 |  | 10.6 |  | Cohen's d |  | -0.0447 |  |
| AT_BrS_7 |  | AT_control_7 |  | Student's t |  | 1.369 |  | 9.00 |  | 0.204 |  | 8.146 |  | 5.95 |  | -5.317 |  | 21.6 |  | Cohen's d |  | 0.4328 |  |
| AT_BrS_8 |  | AT_control_8 |  | Student's t |  | 2.325 |  | 9.00 |  | 0.045 |  | 13.245 |  | 5.70 |  | 0.360 |  | 26.1 |  | Cohen's d |  | 0.7353 |  |
| ARI_BrS_1 |  | ARI_control_1 |  | Student's t |  | -0.126 |  | 9.00 |  | 0.902 |  | -4.558 |  | 36.12 |  | -86.275 |  | 77.2 |  | Cohen's d |  | -0.0399 |  |
| ARI_BrS_2 |  | ARI_control_2 |  | Student's t |  | 1.721 |  | 9.00 |  | 0.119 |  | 34.515 |  | 20.06 |  | -10.857 |  | 79.9 |  | Cohen's d |  | 0.5442 |  |
| ARI_BrS_3 |  | ARI_control_3 |  | Student's t |  | 1.284 |  | 9.00 |  | 0.231 |  | 53.695 |  | 41.82 |  | -40.898 |  | 148.3 |  | Cohen's d |  | 0.4061 |  |
| ARI_BrS_4 |  | ARI_control_4 |  | Student's t |  | 1.049 |  | 9.00 |  | 0.322 |  | 22.373 |  | 21.33 |  | -25.877 |  | 70.6 |  | Cohen's d |  | 0.3317 |  |
| ARI_BrS_5 |  | ARI_control_5 |  | Student's t |  | 0.509 |  | 9.00 |  | 0.623 |  | 15.009 |  | 29.49 |  | -51.710 |  | 81.7 |  | Cohen's d |  | 0.1609 |  |
| ARI_BrS_6 |  | ARI_control_6 |  | Student's t |  | 2.042 |  | 9.00 |  | 0.072 |  | 29.165 |  | 14.28 |  | -3.143 |  | 61.5 |  | Cohen's d |  | 0.6458 |  |
| ARI_BrS_7 |  | ARI_control_7 |  | Student's t |  | 2.444 |  | 9.00 |  | 0.037 |  | 53.011 |  | 21.69 |  | 3.938 |  | 102.1 |  | Cohen's d |  | 0.7728 |  |
| ARI_BrS_8 |  | ARI_control_8 |  | Student's t |  | 0.790 |  | 9.00 |  | 0.450 |  | 26.601 |  | 33.69 |  | -49.601 |  | 102.8 |  | Cohen's d |  | 0.2497 |  |
| RT_BrS_1 |  | RT_control_1 |  | Student's t |  | 0.654 |  | 9.00 |  | 0.529 |  | 29.537 |  | 45.16 |  | -72.617 |  | 131.7 |  | Cohen's d |  | 0.2068 |  |
| RT_BrS_2 |  | RT_control_2 |  | Student's t |  | 1.231 |  | 9.00 |  | 0.250 |  | 42.867 |  | 34.83 |  | -35.918 |  | 121.7 |  | Cohen's d |  | 0.3892 |  |
| RT_BrS_3 |  | RT_control_3 |  | Student's t |  | 2.826 |  | 9.00 |  | 0.020 |  | 66.341 |  | 34.09 |  | 19.228 |  | 173.5 |  | Cohen's d |  | 0.8937 |  |
| RT_BrS_4 |  | RT_control_4 |  | Student's t |  | 2.046 |  | 9.00 |  | 0.071 |  | 62.324 |  | 30.47 |  | -6.594 |  | 131.2 |  | Cohen's d |  | 0.6469 |  |
| RT_BrS_5 |  | RT_control_5 |  | Student's t |  | 0.780 |  | 9.00 |  | 0.455 |  | 20.358 |  | 26.10 |  | -38.689 |  | 79.4 |  | Cohen's d |  | 0.2466 |  |
| RT_BrS_6 |  | RT_control_6 |  | Student's t |  | 0.767 |  | 9.00 |  | 0.463 |  | 21.682 |  | 28.28 |  | -42.287 |  | 85.7 |  | Cohen's d |  | 0.2425 |  |
| RT_BrS_7 |  | RT_control_7 |  | Student's t |  | 0.790 |  | 9.00 |  | 0.450 |  | 29.195 |  | 36.95 |  | -54.392 |  | 112.8 |  | Cohen's d |  | 0.2499 |  |
| RT_BrS_8 |  | RT_control_8 |  | Student's t |  | 0.351 |  | 9.00 |  | 0.733 |  | 8.621 |  | 24.53 |  | -46.867 |  | 64.1 |  | Cohen's d |  | 0.1111 |  |
| Note. Hₐ μ _Measure 1 - Measure 2_ ≠ 0 | | | | | | | | | | | | | | | | | | | | | | | |
|  | | | | | | | | | | | | | | | | | | | | | | | |

**Table 3 Supplementary Materials.** Control patients and BrS patients, both with concomitant RBBB, after ajmaline test. The 7 patients in the BrS group refer to patients with a baseline ECG with RBBB morphology (QRS>120 ms, rSR' in the absence of J-point elevation and ST-T coved segment). As shown in the table, although the ECGs of control patients with RBBB and BrS patients with RBBB may appear similar, the electrophysiological characteristics between the two groups exhibit significant differences. Specifically, RBBB-BrS patients demonstrate an RVa-RVOT ATm and ATm% delay exceeding the cut-off identified in this study (increase% values indicated in bold). In contrast, RBBB-controls have RVOT activation delay below the pathological threshold of 45%.

|  | **RBBB-controls (3)** | **RBBB-BrS (7)** | **P value** |
| --- | --- | --- | --- |
| **anterior-RV, ATm (ms)** | 66,54±8,55 | 68,07±13,08 | 0,86 |
| **RVOT, ATm (ms)** | 95,29±14,48 | 130,36±21,69 | **0,03** |
| **Increase (ms)** | 28,75±16,82 | 62,29±24,26 |  |
| **Increase%** | 43,21%±25,27% | **91,5%±35,6%** |  |
| **anterior-RV, ATm%** | 45,37±7,15 | 45,38±8,82 | 0,92 |
| **RVOT, ATm%** | 64,98±12,57 | 86,91±15,93 | **0,056*** |
| **Increase (ms)** | 19,67±13,51 | 42,34±18,17 |  |
| **Increase%** | 41,27%±29,32% | **92,64%±23,52%** |  |
| **Mean QRS (ms)** | 146,66±14,02 | 150,01±14,71 | 0,75 |

ATm: activation time, mean; BrS: Brugada Syndrome; RBBB: right bundle branch block; anterior-RV: right ventricle, anterior; RVOT: right ventricle outflow tract

**Figure 1 Supplementary Materials.** Panels A, B, C, and D illustrate the baseline ECGs of the patients shown in Figure 1 of the main text.

**
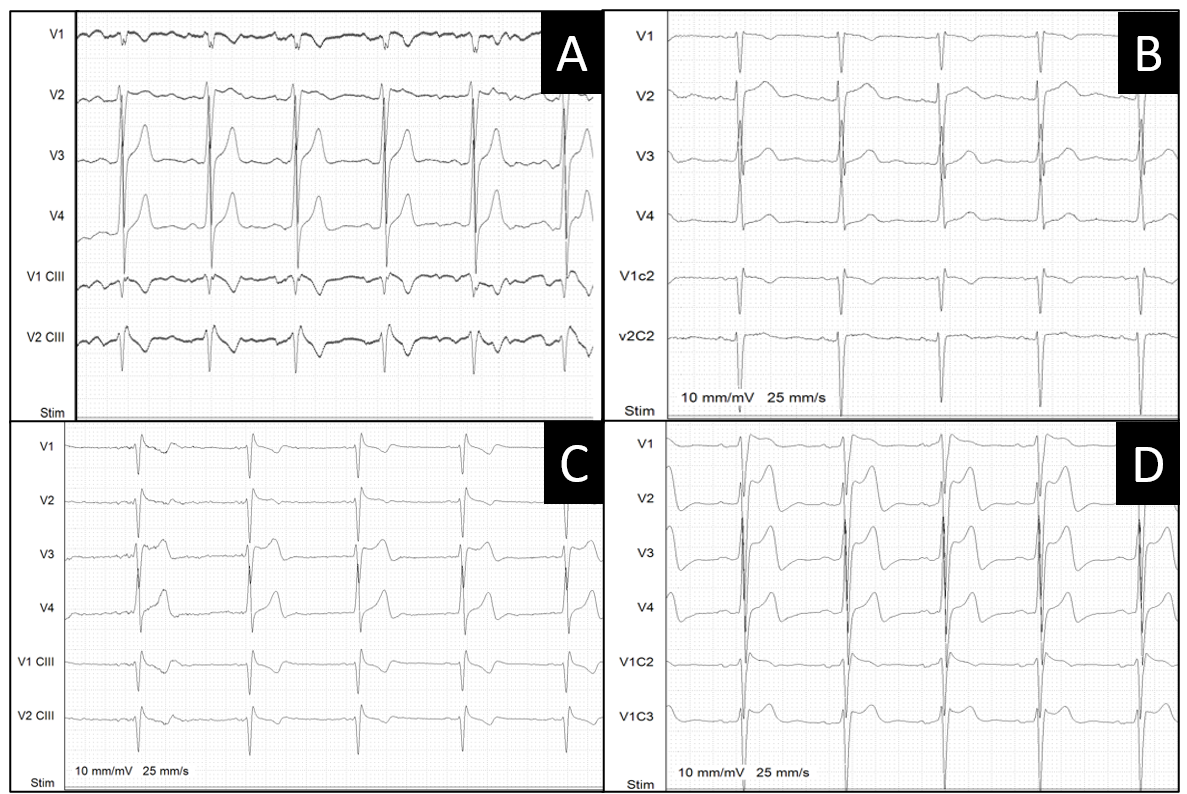
**

**Figure 2 Supplementary Materials.** Workflow of the CardioInsight™ ECGi methodology for non-invasive epicardial mapping.

Panel A: The CardioInsight™ system uses a 252-electrode mapping vest to record body-surface potentials. The vest ensures consistent electrode placement through pre-defined anatomical landmarks. Signals are recorded simultaneously during standard clinical protocols.

Panel B: A low-dose CT scan is performed to construct a patient-specific 3D torso and epicardial model. Electrode positions are projected onto the torso geometry and aligned with the heart’s epicardial surface for accurate inverse reconstruction. The 252-electrode mapping vest captures body-surface ECG potentials.

Panel C: Signal Preprocessing. Raw body-surface ECG signals undergo: 1) Baseline drift correction and 50 Hz noise filtering (Butterworth filter), 2) Beat averaging across three cardiac cycles to enhance signal quality and minimize transient noise, 3) Detection and exclusion of electrodes with poor signal quality using thresholds for impedance and noise levels. The inverse solution uses Tikhonov regularization to reconstruct epicardial potentials, minimizing the amplification of noise from ill-posed equations. The regularization parameter is optimized using the L-curve method. The transfer matrix assumes homogeneous torso conductivity and a static torso-heart model to relate body surface potentials to epicardial nodes.

Panel D: Activation times (ATs) and recovery times (RTs) are annotated using steepest QRS and T-wave slopes, respectively, with outliers removed via percentile-based and spatial filtering.

Panel E: Final AT and RT maps visualize epicardial conduction patterns. On average, 12–15% of electrodes were excluded from the analysis due to poor signal quality. Signals were excluded based on predefined criteria, including high impedance values, excessive noise levels, and unstable baseline drift. The remaining number of usable electrodes across the population was 174 ± 31 (out of 252 electrodes). For areas of interest such as the RVOT, we ensured maximal signal coverage.


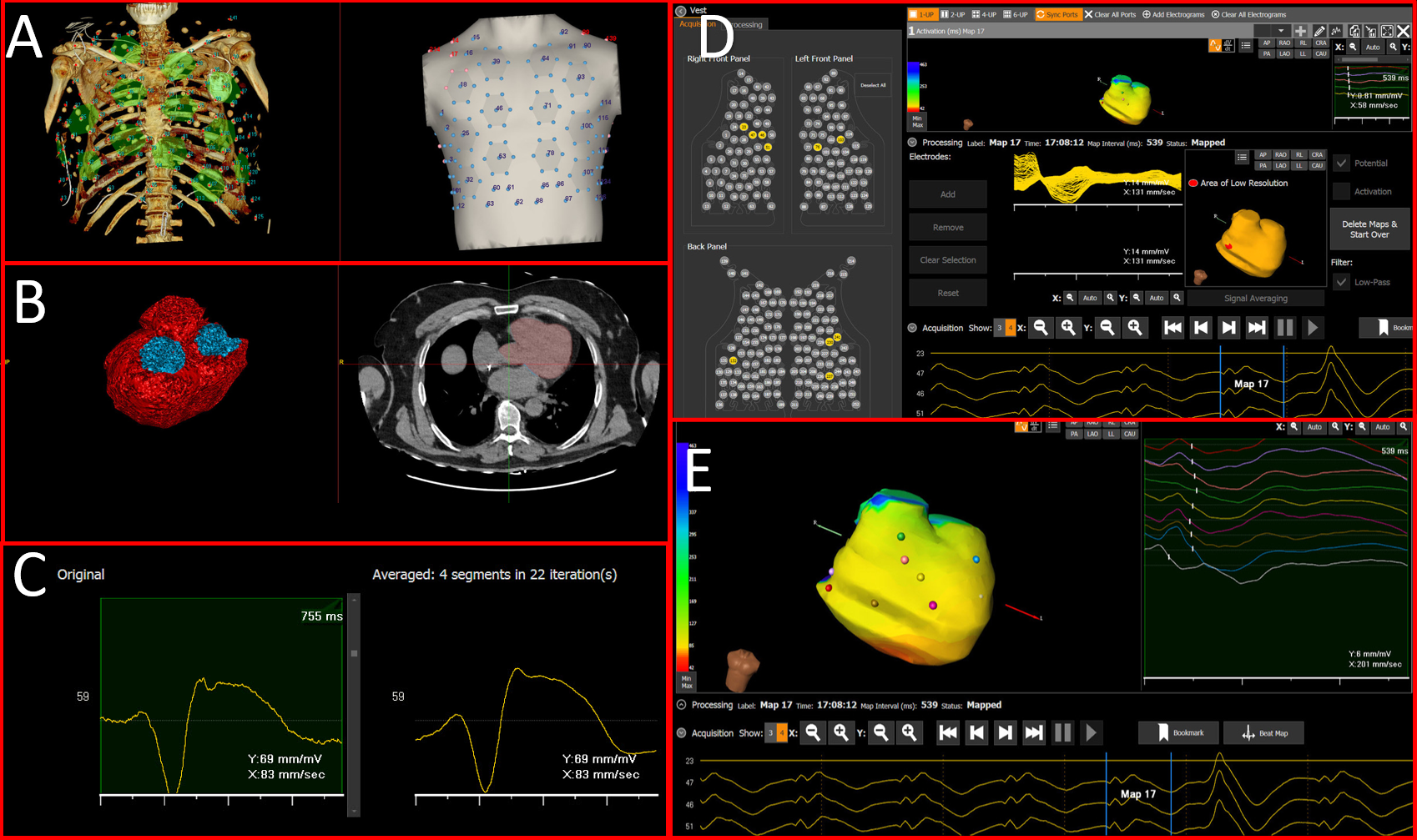


**Figure 3 Supplementary Materials.** The measurement of the mean activation time (ATm) for the RVOT was performed by sampling the entire RVOT region to provide a comprehensive AT value, ensuring stability and minimizing susceptibility to local variations in action potential. As shown in the figure, the entire area was analyzed. The example displayed is from patient 4 in the control group (phase 1). The electrogram (EGM) is highlighted in the box, with its location marked by a yellow point and arrow in the figure. The red points on the EGM indicate the end of the QRS complex and the end of the T wave, annotated by the tool. The red line represents the QRS -dV/dT, also automatically annotated by the tool.


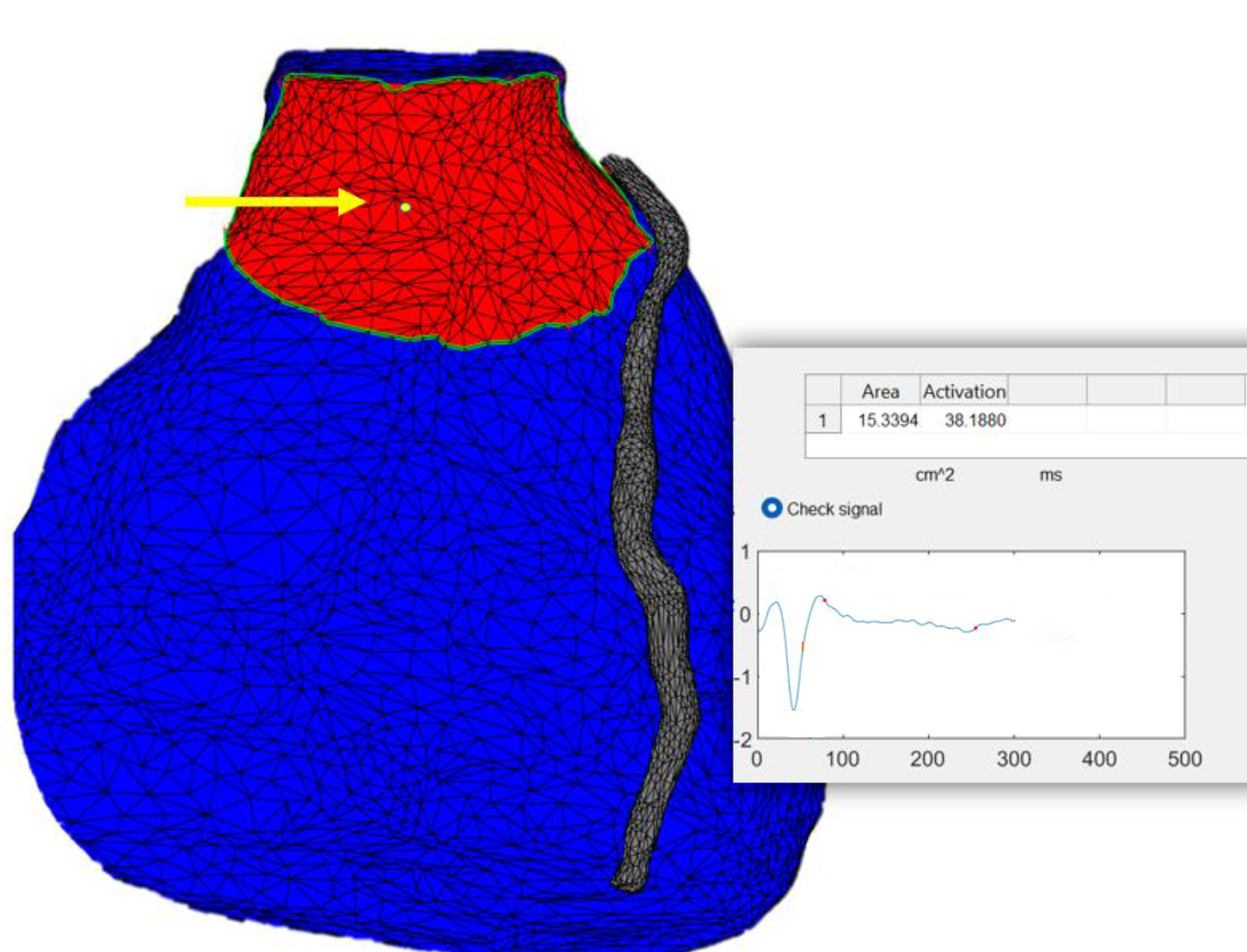


**Figure 4 Supplementary Materials.** ATm, ARIm and RTm distributions in BrS pattern-positive and control group of the Phase 1.

1: anterior RV; 2: RV apex; 3: RVOT; 4: posterior-lateral RV; 5: anterior LV; 6: LV apex; 7: LVOT; 8: posterior-lateral LV.


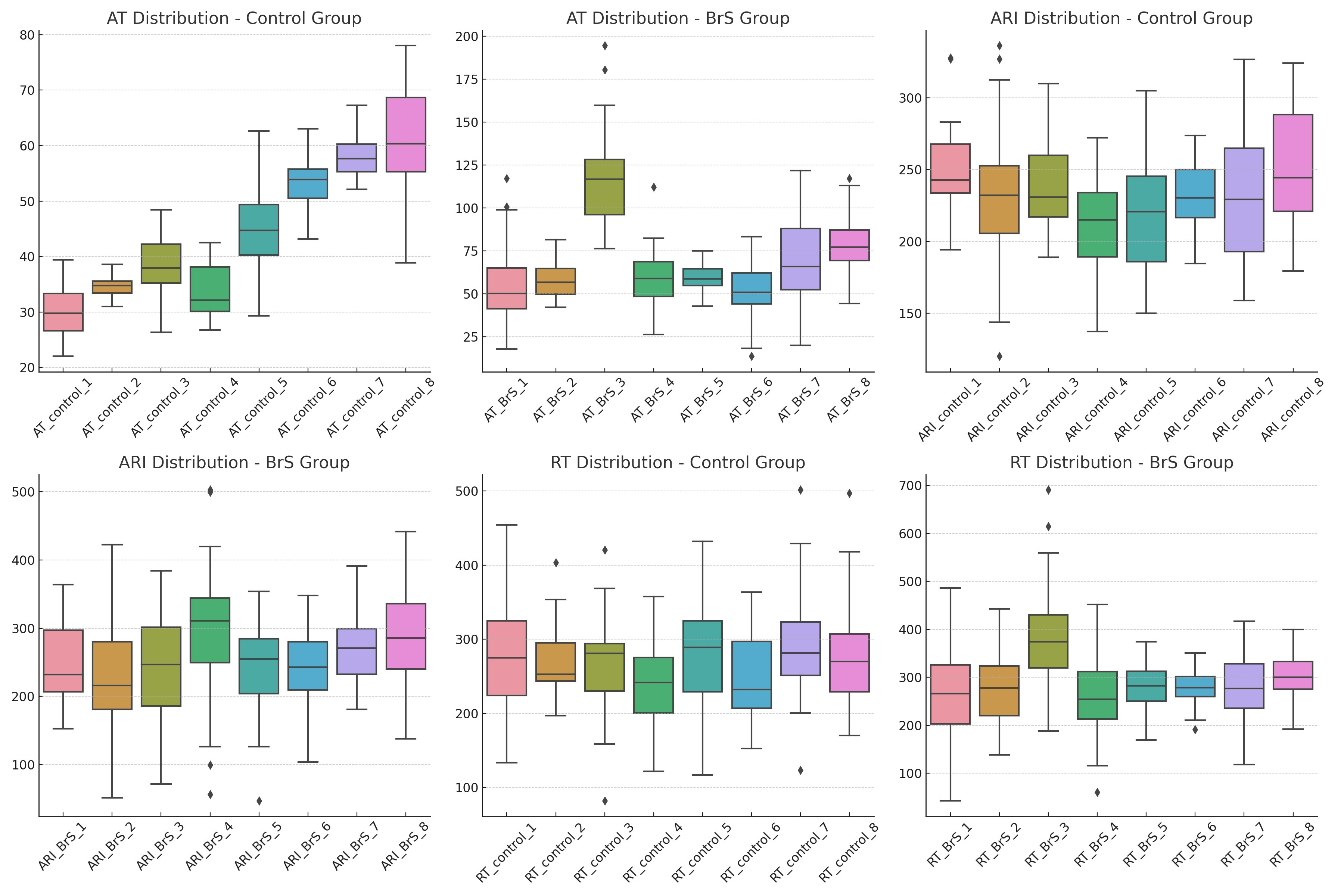


### BrS first breakthrough

Normal human ventricular epicardial activation during sinus rhythm (SR) appears in the anterior RV, as have been reported previously^1–3^. The comparison of the first breakthrough between controls and BrS groups did not show differences both in absence or presence of pattern 1 ECG (controls vs BrS pattern-negative: 26,1±3,81 vs 28,62±4,79 ms, p=0,12; controls vs BrS pattern-positive: 26,1±3,81 vs 29,08±4,59 ms, p=0,057), while a difference was noted for the RV breakthrough in controls vs RBBB patients (26,1±3,81 vs 55,33±6,77 ms, p=0,001).

Both in our study and in previous, it emerges that the activation in BrS patients occurs first in the anterior aspect of RV, but with a non-negligible delay compared to the normal activation times of a healthy patient^2^ (52±12 msec, Zhang and colleagues, Circulation 2015^4^). Prior to activation spreading to the epicardial surface of the anterior-RV, the emergency of the first breakthrough in BrS occurs without any delay compared to controls. In contrast, a significant difference was noted for controls and RBBB patients (26,1±3,81 vs 55,33±6,77 ms; p=0,001), indicating that in BrS there is no delay of activation in the conduction tissue but this begins after the endo-epicardial Purkinje fibres propagation, when the conduction spreads in the epicardium. In fact, in endocardial studies conducted on BrS patients, the first activation in the RV, although delayed compared to controls, is maintained at a nonsignificant delay value (31 msec vs 17 msec^5^). In patients with RBBB the emergence of the first breakthrough in the RV is less consistent and occurs over a longer period of time. This is because the activation of the RV is secondary to that of the left ventricle (LV)^6^, but considering that the conduction defect is found within the conducting tissue, the emergence of the I BT is delayed compared to controls and BrS patients (BrS pattern-negative vs RBBB: 28,62±4,79 ms vs 55,33±6,77 ms, p<0,001; BrS pattern-positive vs RBBB: 29,08±4,59 ms vs 55,33±6,77 ms, p<0,001).

1. Durrer D, van Dam RT, Freud GE, Janse MJ, Meijler FL, Arzbaecher RC. Total excitation of the isolated human heart. Circulation 1970;41. doi:10.1161/01.CIR.41.6.899.

2. Ramanathan C, Jia P, Ghanem R, Ryu K, Rudy Y. Activation and repolarization of the normal human heart under complete physiological conditions. Proc Natl Acad Sci U S A 2006;103. doi:10.1073/pnas.0601533103.

3. Wyndham CR, Meeran MK, Smith T, Saxena A, Engelman RM, Levitsky S, et al. Epicardial activation of the intact human heart without conduction defect. Circulation 1979;59. doi:10.1161/01.CIR.59.1.161.

4. Zhang P, Tung R, Zhang Z, Sheng X, Liu Q, Jiang R, et al. Characterization of the epicardial substrate for catheter ablation of Brugada syndrome. Heart Rhythm 2016;13. doi:10.1016/j.hrthm.2016.07.025.

5. Postema PG, van Dessel PFHM, de Bakker JMT, Dekker LRC, Linnenbank AC, Hoogendijk MG, et al. Slow and discontinuous conduction conspire in Brugada syndrome: a right ventricular mapping and stimulation study. Circ Arrhythm Electrophysiol 2008;1. doi:10.1161/CIRCEP.108.790543.

6. Ramanathan C, Ghanem RN, Jia P, Ryu K, Rudy Y. Noninvasive electrocardiographic imaging for cardiac electrophysiology and arrhythmia. Nat Med 2004;10. doi:10.1038/nm1011.
